# Suppression of the coffee-ring effect by sugar-assisted depinning of contact line

**DOI:** 10.1038/s41598-018-35998-w

**Published:** 2018-12-11

**Authors:** Shunsuke F. Shimobayashi, Mikiko Tsudome, Tomo Kurimura

**Affiliations:** 1 Department of Mathematical Science and Advanced Technology, Japan Agency for Marine-Earth Science and Technology (JAMSTEC), Yokohama, 236-0001 Japan; 2 Research and Development Center for Marine Biosciences, Japan Agency for Marine-Earth Science and Technology (JAMSTEC), Yokosuka, 237-0061 Japan; 3 Institute of Innovative Research, Tokyo Institute of Technology, Yokohama, 226-8503 Japan

## Abstract

Inkjet printing is of growing interest due to the attractive technologies for surface patterning. During the printing process, the solutes are transported to the droplet periphery and form a ring-like deposit, which disturbs the fabrication of high-resolution patterns. Thus, controlling the uniformity of particle coating is crucial in the advanced and extensive applications. Here, we find that sweet coffee drops above a threshold sugar concentration leave uniform rather than the ring-like pattern. The evaporative deposit changes from a ring-like pattern to a uniform pattern with an increase in sugar concentration. We moreover observe the particle movements near the contact line during the evaporation, suggesting that the sugar is precipitated from the droplet edge because of the highest evaporation and it causes the depinning of the contact line. By analyzing the following dynamics of the depinning contact line and flow fields and observing the internal structure of the deposit with a FIB-SEM system, we conclude that the depinned contact line recedes due to the solidification of sugar solution without any slip motion while suppressing the capillary flow and homogeneously fixing suspended particles, leading to the uniform coating. Our findings show that suppressing the coffee-ring effect by adding sugar is a cost-effective, easy and nontoxic strategy for improving the pattern resolution.

## Introduction

When a drop of coffee evaporates on a solid substrate, a ring-like stain is often observed, which is called a ‘coffee-ring’. If the contact line of an evaporating drop is fixed, the fluid flux towards the edge of the drop is driven by surface tension, which carries suspended particles to the edge, leading to a ring-like deposit^1–4^. This outward capillary flow disrupts the uniformity of the deposit, which is problematic for the advanced and extensive applications of inkjet technologies^5–10^. Therefore, controlling the deposit pattern is the key issue and numerous studies have been devoted to the suppression of the coffee-ring effect with electrowetting^11^, high-aspect-ratio particles^12,13^, evaporation speeds^14^, photosensitive surfactants^15,16^, and various mixtures of surfactants and polymers^17^; however, our knowledge is still limited regarding costs, simplicity, and invasiveness^1^.

**Figure 1:**
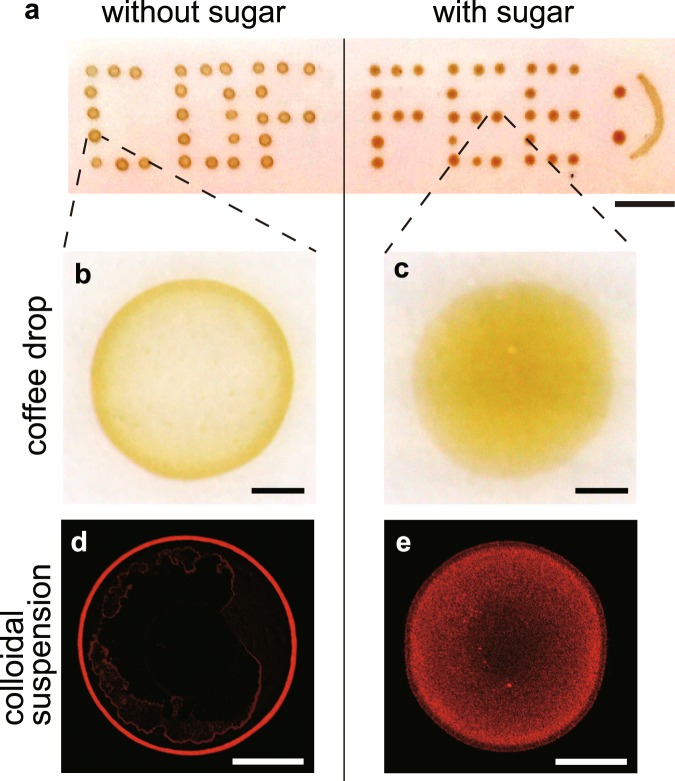
Evaporative deposits. (**a**) Bright field image of coffee drops without sugar and with caster sugar (97.8% sucrose) in 200 mM. The scale bar is 1 cm. (**b**,**c**) Enlarged images from (**a**). The scale bars are 500 *μ*m. (**d,e**) Confocal images of the final depositions of colloidal drops (1 *μ*m in diameter, 0.5 *μ*l, 0.1 vol%) without sugar (**d**) and with sucrose in 200 mM (**e**). The scale bars are 500 *μ*m.

Here, we focus on the effects of sugar on the deposition pattern of suspended particles. We find that sweet coffee drops above a threshold sugar concentration leave uniform rather than ring-like stains after evaporation on a solid surface (Fig. 1), which inspired us to investigate the coating mechanism using colloidal suspensions that mimic coffee drops. We experimentally demonstrate that the uniform pattern is due to the interplay of the contact line behaviours and particle adhesion on the surface, both are altered by sugar precipitation from the droplet edge.

## Results and Discussion

Boiled water at a temperature of approximately 80 °C was poured into ground coffee powders to make a brewed coffee. When a coffee drop (0.5 *μ*L) was evaporated on a solid substrate (here pre-cleaned ceramic dish plate), a ring-like stain was observed (Fig. 1a,b). During the evaporation, the temperature and relative humidity were fixed, i.e., *T* = 298 ± 2 K and *RH* = 40 ± 3%. The particle size was approximately 100 nm in radius using dynamic light scattering (Supplementary Fig. S1). In contrast, when sugar (caster sugar, ~98% sucrose) was used to moderately sweeten the coffee (a 0.5 *μ*L coffee drop with a sugar concentration of 200 mM), no ring-like stains were observed, and the stains were somewhat uniform (Fig. 1a,c).

**Figure 2:**
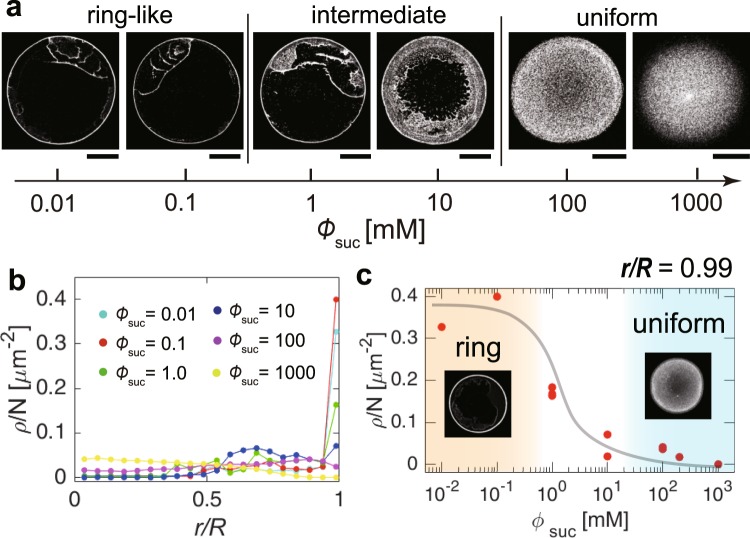
Deposit pattern change as a function of sucrose concentration. (**a**) Evaporative deposits of drops (0.5 *μ*L, 0.01 vol%) with ${\varphi }_{{\rm{suc}}}={10}^{-2},{10}^{-1},{10}^{0},{10}^{1},{10}^{2}$ and 10^3^ mM. The ring-like pattern transits to the uniform pattern through the intermediate pattern. The scale bars are 500 *μ*m. (**b**) Normalized particle number density, *ρ*(*r*)/*N*, as a function of normalized radial distance, *r*/*R*, from the deposit centre for drops with ${\varphi }_{{\rm{suc}}}={10}^{-2},{10}^{-1},{10}^{0},{10}^{1},{10}^{2}$ and 10^3^ mM. (**c**) Normalized particle number density near the edge (*r*/*R* = 0.99) as a function of ${\varphi }_{{\rm{suc}}}$. The black line is a visual guide.

To make the particle distribution clear, we used colloidal suspensions of fluorescent polystyrene (580/605 nm, 1 *μ*m in diameter) that mimic coffee drops. When a drop (0.5 *μ*L, 0.1 vol%) without sucrose was evaporated, a ring-like deposit was observed under a fluorescent microscope (Fig. 1d). In contrast, when a drop with a sucrose concentration of 200 mM was evaporated, the particles exhibited a uniform distribution (Fig. 1e). Investigating the patterns as a function of sucrose concentration from 10^−2^ to 10^3^ mM revealed that the ring-like pattern changed to an uniform pattern through an intermediate pattern as sucrose concentration ${\varphi }_{{\rm{suc}}}$ increases (Fig. 2a). To quantitatively investigate the change, we estimated the areal particle number density, *ρ*(*r*), as a function of radial distance, *r*, from the droplet centre for drops with ${\varphi }_{{\rm{suc}}}={10}^{-2},{10}^{-1},{10}^{0},{10}^{1},{10}^{2}$ and 10^3^ mM (Fig. 2b). The particle density *ρ*(*r*) and radial distance *r* are normalized by the total particle number *N* and the deposit radius *R*, respectively. For drops with low sucrose concentration (${\varphi }_{{\rm{suc}}}$ = 10^−2^ and 10^−1^ mM), the density profile has a large peak, *ρ*(*r*)/*N* ~ 0.4, at *r*/*R* ~ 1. For the drops with a medium sucrose concentration (${\varphi }_{{\rm{suc}}}$ = 10^0^ mM), the profile has a smaller peak, *ρ*(*r*)/*N* ~ 0.2. In contrast, for drops with a high sucrose concentration (${\varphi }_{{\rm{suc}}}$ = 10^1^, 10^2^ and 10^3^ mM), the profile does not have any peak. Rather, for ${\varphi }_{{\rm{suc}}}={10}^{3}$ mM, the density appears higher at the centre. As ${\varphi }_{{\rm{suc}}}$ increases, the final deposit becomes thicker. As a result, for ${\varphi }_{{\rm{suc}}}={10}^{3}$ mM, the periphery in the image is out of focus (Fig. 2a), leading to the underestimate of the particle density near the periphery. The change in particle density near the edge, *r*/*R* = 0.99, is summarized in Fig. 2c.

To elucidate the mechanism behind the pattern change, we investigated the evaporation dynamics. We first quantified the initial shapes of drops characterized by the maximum height *h*_0_, radius *R*, and contact angle *θ* (Supplementary Fig. S2). For ten drops with ${\varphi }_{{\rm{suc}}}$ = 0 and 200 mM, these parameters were estimated by the shape analysis to be (*h*_0_, *R*, *θ*) = (325 ± 19 *μ*m, 876 ± 36 *μ*m, 35.2 ± 1.8°) for ${\varphi }_{{\rm{suc}}}$ = 0 mM and (*h*_0_, *R*, *θ*) = (311 ± 10 *μ*m, 857 ± 25 *μ*m, 34.7 ± 2.8°) for ${\varphi }_{{\rm{suc}}}$ = 200 mM. This similarity of the parameter values ensures that the initial shapes are independent of ${\varphi }_{{\rm{suc}}}$. Furthermore, we estimated the evaporation speed from the mass change of drops for ${\varphi }_{{\rm{suc}}}$ = 0 and 200 mM, respectively. The evaporation speed is almost constant in time (−100 *μ*g/s, Supplementary Fig. S3). Regardless of the similar initial shapes and evaporation rates, the deposit pattern of particles completely changes as a function of ${\varphi }_{{\rm{suc}}}$.

**Figure 3:**
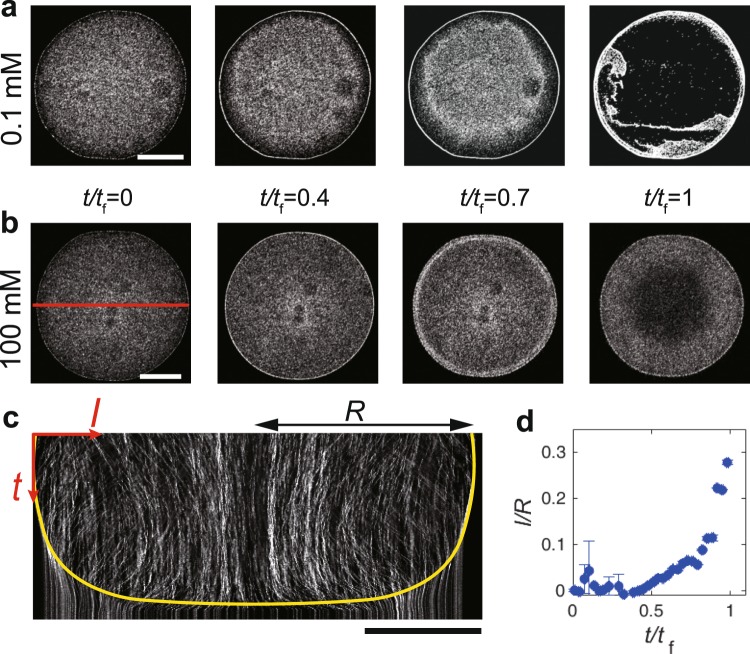
(**a**,**b**) Sequential fluoresce images near the bottom during the evaporation of a drop (0.5 *μ*l, 0.01 vol%) of particle suspension with ${\varphi }_{{\rm{suc}}}$ = 0.1 and 100 mM. The scale bars are 500 *μ*m. (**c**) Spatio-temporal image of the red-solid line in (**b**). The yellow line is the eye guide of the receding front toward the center. The particles do not move below the line. The scale bars are 500 *μ*m. (**d**) Distance of the receding front from the droplet edge normalized by the droplet radius, *l*/*R*, as a function of normalized time, *t*/*t*_f_, where *l* and *R* are the distance of the receding front from the edge and the droplet radius, respectively.

**Figure 4:**
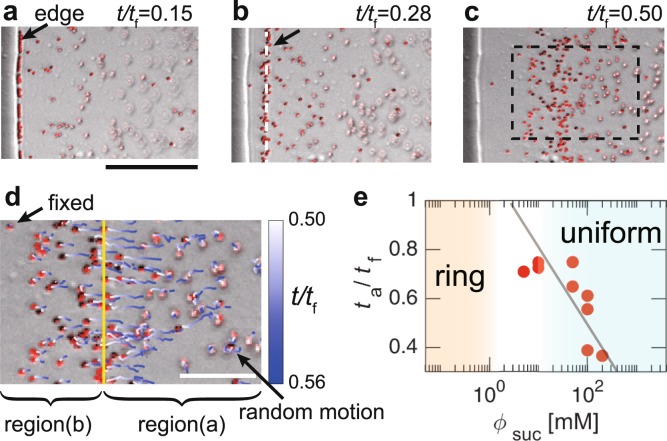
(**a**–**c**) Sequential images near the contact line of a drop with ${\varphi }_{{\rm{suc}}}$ = 100 mM at *t*/*t*_f_ = (**a**) 0.15, (**b**) 0.28 and (**c**) 0.50, where *t*_f_ is the total evaporation time. The fluoresce images are merged with bright-field ones. The scale bar is 50 *μ*m. The arrow and white dashed line in (**b**) show the front of the particles that move towards the centre from the edge. The arrow in (**a**) indicates the edge of the solid-like sucrose. (**d**) Particle trajectories are shown by time-dependent colour lines in the rectangle in (**c**) from *t*/*t*_f_ = 0.50 to *t*/*t*_f_ = 0.56. The yellow line indicates the front. Regions (**a** and **b**) show the inner and outer regions divided by the front, respectively. The scale bar is 20 *μ*m. (**e**) The normalized time at which the particles start to move inward from the edge, *t*_a_/*t*_f_, as a function of ${\varphi }_{{\rm{suc}}}$. The line is a visual guide.

Sequential fluoresce images of an evaporating drop with ${\varphi }_{{\rm{suc}}}$ = 0.1 mM reveal that the particles gradually accumulate at the edge and form a ring-like deposit, as observed in the common coffee ring (Fig. 3a, Supplementary Movie 1). In contrast, for a drop with ${\varphi }_{{\rm{suc}}}$ = 10^2^ mM, the particles seem to stop accumulating at *t*/*t*_f_ ~ 0.4 and the front moving toward the center was immediately observed, where *t*_f_ (~200 s) is the total evaporation time (Fig. 3b, Supplementary Movie 2). To see the receding front, we investigated spatio-temporal dynamics of the red solid line in Fig. 3b (Fig. 3c). We moreover evaluated the front dynamics from the time difference analysis of the intensity and found that the particles are motionless behind the front. We also found that the velocity of the receding front is nearly zero till *t*/*t*_f_ ~ 0.4, is kept constant (~0.5 *μ*m/s) over $0.4\lesssim t/{t}_{{\rm{f}}}\lesssim 0.8$, and then greatly increases (Fig. 3d). To observe the onset of the receding event more clearly, the particle behaviour near the contact line was magnified (Fig. 4a–d). Time-resolved fluorescence images (merged with bright-field images) reveal that the accumulated particles at the edge start to move towards the droplet centre at a certain time, which is denoted by *t*_a_ (Fig. 4a,b, Supplementary Movie 3). Even after *t* = *t*_a_, the particles at the edge continued to move towards the centre, while some were left behind the front. As shown in Fig. 4e, *t*_a_ increases as ${\varphi }_{{\rm{suc}}}$ decreases, as shown by the black line. The particles at the edge did not move for values of ${\varphi }_{{\rm{suc}}}\lesssim 1\,{\rm{mM}}$ (indicated by the orange colored region).

For the further investigation, we tracked the particle motion in the rectangle region of Fig. 4c from *t*/*t*_f_ = 0.50 to *t*/*t*_f_ = 0.56 (here, *t*_a_/*t*_f_ = 0.22) as shown with time-dependent colour lines (Fig. 4d). At *t*/*t*_f_ = 0.5, the front (indicated by the yellow line) moved inward with a velocity (~0.5 *μ*m/s). The particles in region (a) seemed to be above the focal plane and underwent random motion in the solution. However, in region (b), some particles were fixed, while others slightly move towards the centre and immediately became fixed. The motionless particles must coexist with solid-like sucrose because the edge (indicated by the black arrow in Fig. 4a) is still remained even after the complete evaporation of water. The physical states of regions (a) and (b) are therefore considered to be liquid-like and solid-like, respectively, suggesting that the solid-like sucrose, which may be an amorphous state, emerged from the edge because of the highest evaporation near the contact line^2,3^. Then, it should have caused the depinning of the glass-liquid-gas contact line, as suggested by the theoretical studies on the drying process of polymer solutions^18,19^. Thus, the moving front should correspond to the solid sucrose-liquid-gas boundary (hereafter called SLG boundary) depinned from the glass^18^. This front has subsequently passed towards the centre with the homogeneous fixation of the particles. This process breaks the ring-like structure of the particles and homogeneously disperses the particles, contributing to the uniformity of the particle coating.

**Figure 5:**
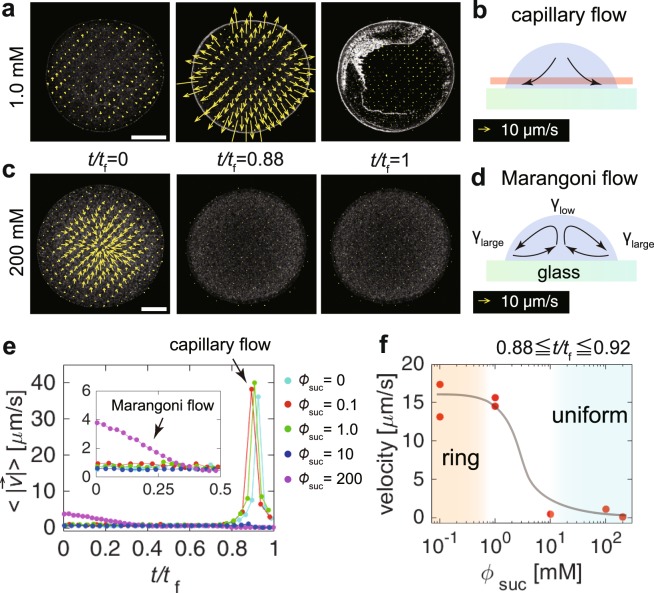
(**a**,**c**) Flow fields of drops (0.5 *μ*l, 0.01 vol%) with ${\varphi }_{{\rm{suc}}}$ = (**a**) 1.0 and (**c**) 200 mM. The scale bars are 500 *μ*m. (**b**,**d**) Schematic of (**b**) the evaporatively driven capillary flow and (**d**) the sugar driven solutal Marangoni flow. The arrows represent the flow pattern. The analysis was done near the substrate as shown by the red-shaded region. (**e**) The velocity magnitude averaged over the drops (${\varphi }_{{\rm{suc}}}$ = 0, 0.1, 1, 10 and 200 mM) as a function of normalized time *t*/*t*_f_. The arrow indicates the capillary flow that rapidly grows at *t*/*t*_f_ ~ 0.9. The inset shows the first half and the arrow in the inset shows the Marangoni flow driven by the surface-inactive sucrose in a drop with ${\varphi }_{{\rm{suc}}}$ = 200 mM. (**f**) The average velocity in the time span $0.88\,\leqq \,t/{t}_{{\rm{f}}}\,\leqq \,0.92$ as a function of ${\varphi }_{{\rm{suc}}}$. The black line is a visual guide.

If the depinning of the contact line occurs at *t* = *t*_a_, then the capillary flow can be suppressed^1,11,20^. To test the hypothesis, we determined the entire flow pattern by performing particle image velocimetry (PIV) analysis^21^. For drops with low sucrose concentration (${\varphi }_{{\rm{suc}}}$ = 0, 0.1 and 1.0 mM), the outward radial capillary flow rapidly increased at *t*/*t*_f_ ~ 0.9 (Fig. 5a,b,e, and Supplementary Movie 4). When the averaged velocity magnitude, $\langle |\overrightarrow{v}(t)|\rangle$, reaches a maximum (~40 *μ*m/s), the depinning of the contact line occurs. However, the ring-like structure of the particles does not move due to shear stresses caused by the depinning, thus maintaining the ring-like particle distribution. However, for drops with a high sucrose concentration (${\varphi }_{{\rm{suc}}}$ = 10 and 200 mM), the growth of the capillary flow at *t*/*t*_f_ ~ 0.9 was not observed (Fig. 5e). To quantitatively compare the flow, we calculated the average velocity magnitude within a range of $0.88\,\leqq \,t/{t}_{{\rm{f}}}\,\leqq \,0.92$ for drops with ${\varphi }_{{\rm{suc}}}$ = 0.1, 1.0, 10, 100 and 200 mM. As shown in Fig. 5f, it was confirmed that the capillary flow is suppressed above a threshold concentration ${\varphi }_{{\rm{suc}}}^{\ast }\sim 10$ mM. The results ensure that the depinning occurs at *t* = *t*_a_ when ${\varphi }_{{\rm{suc}}}\gtrsim {\varphi }_{{\rm{suc}}}^{\ast }$.

At the initial stage of evaporation, we observed a vortex with an inward (outward) flow at the bottom (top) when ${\varphi }_{{\rm{suc}}}\gtrsim 100\,{\rm{mM}}$ (Fig. 5c–e, Supplementary Fig. S4, Supplementary Movies 5 and 6), which is the solutal Marangoni flow driven by the variable concentrations of surface-inactive sucrose at the liquid-air interface^22^. The mean velocity for ${\varphi }_{{\rm{suc}}}$ = 200 mM is ~4 *μ*m/s, and gradually decreases to nearly zero at *t*/*t*_f_ ~ 0.4 (see the inset in Fig. 5e). However, such a Marangoni flow was not observed when ${\varphi }_{{\rm{suc}}}$ = 10 mM although the particles are homogeneously fixed. Therefore, the uniform coating is not caused by the Marangoni flow although it has often been reported that Marangoni flow can redistribute the particles at the edge of the droplet towards the inside^17,23–26^.

After the contact line of a liquid droplet is depinned on a solid substrate, the sliding motion is often confirmed^27^. However, such a sliding motion was never observed in our system, suggesting that the movement of the SLG boundary toward the droplet center is not such a sliding motion. We moreover do not observe the emergence of the internal flow, which is assumed if the movement of the SLG boundary is a sliding motion (Fig. 5e). We therefore consider that the movement is a passive motion driven by the solidification of sucrose solution from the edge (Fig. 3c,d), as suggested by the theoretical studies^18^.

**Figure 6:**
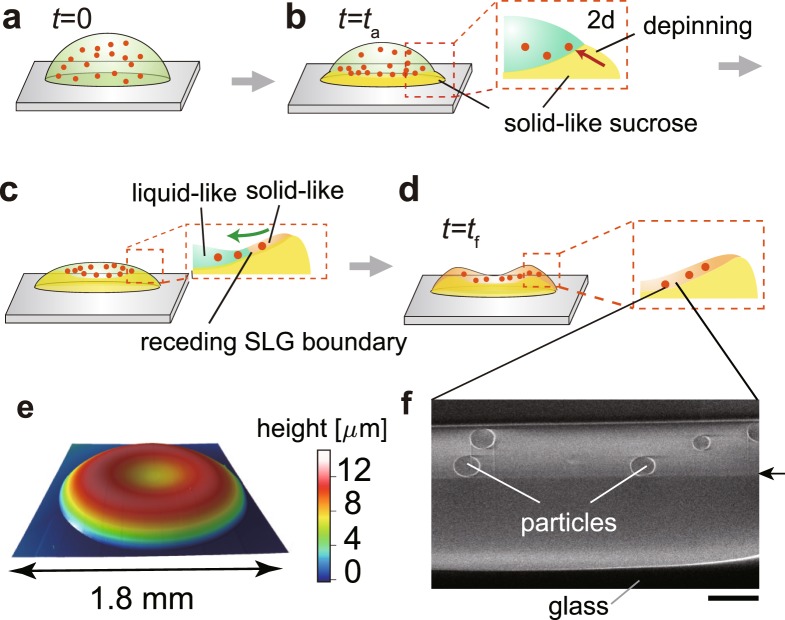
(**a**–**d**) Schematic of the evaporation process that leads to a uniform particle coating. (**e**) Height profile of the final deposit of a drop with ${\varphi }_{{\rm{suc}}}$ = 200 mM. (**f**) SEM image of a cross section of an evaporative deposit with ${\varphi }_{{\rm{suc}}}$ = 200 mM sliced by focused ion beam (FIB). The arrow indicates the horizontal line. The particles exist only in the upper layer. The scale bar is 2 *μ*m.

To investigate the internal structure of the final deposit, we performed focused ion beam-scanning electron imaging for a drop with ${\varphi }_{{\rm{suc}}}$ = 200 mM. Interestingly, we found a horizontal line in a vertical cross-sectional image of the deposit for ${\varphi }_{{\rm{suc}}}$ = 200 mM (Fig. 6d,f), which is indicated by the arrow in Fig. 6f (Supplementary Fig. S5 for a wider view). The particles were confined only above the line, and thus, the materials below the line are exclusively sucrose. The line should provide evidence of solidification of liquid solution that contains particles on the solid-like sucrose (Fig. 6c). We moreover studied the shape of the final deposit with optical profilometer and found that it has a shallow dent with the centre (~5 *μ*m), which has been observed in drying films of polymer solutions as well^18,19^ (Fig. 6e). The shape with a shallow dent can be attributed to the dramatic increase of the receding speed of the SLG boundary at the late stage of evaporation, i.e., $t/{t}_{{\rm{f}}}\gtrsim 0.8$, (Fig. 3c,d).

We now discuss the underlying mechanism behind the homogeneous deposit of particles for the droplets with ${\varphi }_{{\rm{suc}}}$ = 100–1000 mM. The sucrose is precipitated from the droplet edge because of the highest evaporation, resulting in the depinning of the contact line, simultaneously the emergence of the SLG boundary, and the suppression of the capillary flow. The SLG boundary recedes due to the solidification of liquid solution that contains particles on solid-like sucrose prepared on the glass substrate without any slip motion. Moreover, the dramatic increase of the receding speed at the late stage, i.e., $t/{t}_{{\rm{f}}}\gtrsim 0.8$, may lead to the final shape of the deposit with a shallow dent at the center (Fig. 6). As shown in Fig. 2a, the droplets with ${\varphi }_{{\rm{suc}}}$ = 1–10 mM leave an intermediate pattern, which can be attributed to the termination of the recession due to the absence of the sucrose (Supplementary Movie 7).

Finally, to examine the universality of our findings, we studied the deposit patterns of drops that contain glucose or fructose instead of sucrose and confirmed that they also exhibited uniform deposits (Supplementary Fig. S6). Moreover, it has recently been reported that colloidal suspensions that contain hydrosoluble polymers^28^ or salts^29^ leave the uniform pattern above a threshold concentration. Exploring the common physicochemical properties of the additives to suppress the coffee ring effect would be useful in further understanding the underlying mechanism.

The physical strategies for suppressing the coffee ring effect can be classified into three categories^1^ (i) occurrence of the depinning of the contact line^11,20^; (ii) disturbing the capillary flow using different flows, such as Marangoni^23–26^ or electro-osmotic flow^30^; (iii) prevention of the particles being transported to the edge by the capillary flow^13–16^. The mechanism in our system belongs to the first category, i.e., the suppression by the depinning of the contact line, but the particle coating process is distinct from previously described processes^1,11,20^. In contrast to them, the particles are coated behind the receding SLG boundary, which thus enables the determination of the region to be coated initially. Generally, expensive external devices^30^, multi-step procedures^20^ and organic solvents^26^ are required for controlling the uniformity of the deposit. We have revealed that the uniformity can be controlled only by adding sugar. This cost-effective, easy and non-toxic method for obtaining uniform deposits would thus be useful in many areas of engineering.

## Methods

### Evaporative deposits of non-sweet and sweet coffee drops

Coffee beans were “Azabu blend” bought from Azabu Kobo in Japan. 240 mL of boiled water at around 80 °C was poured into 20 g of the coffee powders in a virgin pulp coffee filter (HARIO, Japan). To make a sweet coffee, 4 g of white caster sugar (Nissin Sugar Co., Ltd., Japan) was added to 60 mL of coffee. As 97.8% of the sugar is sucrose, the sucrose concentration is about 200 mM. The drop (0.5 *μ*L) was evaporated on a ceramic dish plate (Lasagna dish L, Aeon, Japan) pre-cleaned by water. During the evaporation, temperature and relative humidity were kept within 298 ± 5 K and 30 ± 5%, respectively. The evaporative deposits were imaged by a Olympus SZXV upright microscope with SZ2-LGB illuminator and acquired by a camera (NY-X6i, Canon). The length was measured using a calibrated ocular micrometer (OBM1/100, Olympus).

### Evaporative deposits of non-sweet and sweet colloidal drops

Carboxylated-modified fluorescent polystyrene particles (580/605 nm, 1 *μ*m in diameter, 2 vol%) were bought from Invitrogen (USA). To suspend the colloids in pure water, the solution was centrifuged at 3000 × g at 25 °C for 15 min, and the supernatant was replaced by Milli-Q (18.2 MΩ cm) to remove the surfactants. The procedure was repeated three times. The colloidal solution was diluted in 0.1 or 0.01 vol% with Milli-Q (18.2 MΩ cm). The diluted drop (0.5 *μ*L) was evaporated on a soda-lime glass with 8 reaction wells (Marienfeld, Germany), which were pre-cleaned by Milli-Q (18.2 MΩ cm) and ethanol before use. During the evaporation, temperature and relative humidity were kept within 298 ± 5 K and 30 ± 5%, respectively. The evaporative deposits were imaged on a inverted microscope (Nikon Ti-E, Japan) with a confocal laser scanning system (Nikon A1, Japan), equipped with ×4, 0.13 NA and ×20, 0.75 NA dry objective lens (Nikon, Japan).

### Particle density analysis in evaporative deposits

We first removed noise from raw images using the 2D median filter with a window size 3 × 3 pixels. Then, we fitted the edge of an evaporative deposit by an ellipse and determined the centre position. The drop radius *R* was estimated to be (*a* + *b*)/2, where *a* and *b* are long and short axis lengths of the fitted ellipse, respectively. By assuming that the particle number per unit area is linearly dependent on the fluorescent intensity per unit area, the particle density *ρ*(*r*) (0 ≤ *r* ≤ *R*) was defined in the continuous limit as a function of radial distance from the centre as follows;

1 $$\begin{array}{rcl}\rho (r) & \equiv  & \mathop{\mathrm{lim}}\limits_{dr\to 0}\,\frac{\alpha \,{\int }_{r-dr/2}^{r+dr/2}\,{\int }_{0}^{2\pi }\,I(r^{\prime} ,\theta )r^{\prime} dr^{\prime} d\theta }{{\int }_{r-dr/2}^{r+dr/2}\,{\int }_{0}^{2\pi }\,r^{\prime} dr^{\prime} d\theta }\\  & = & \mathop{\mathrm{lim}}\limits_{dr\to 0}\,\frac{\alpha }{\pi {r}^{2}}\,{\int }_{r-dr/2}^{r+dr/2}\,{\int }_{0}^{2\pi }\,I(r^{\prime} ,\theta )r^{\prime} dr^{\prime} d\theta \end{array}$$

where *α* is the constant of proportionality, and *I*(*r*, *θ*) is the fluorescent intensity at a point (*r*, *θ*). The particle density was calculated in the region [*r* − *dr*/2, *r* + *dr*/2], where *dr* is *R*/80.

### Flow filed analysis in evaporating drops

Motion of fluid flows in an evaporating droplet was studied by particle image velocimetry (PIV). First, we recorded the sequential confocal images at a certain height and masked the areas except for the inside of the droplet. The region of interest was divided into squares with a side of 64 pixels and calculated the velocity field at each centre using the direct Fourier transform correlation. This calculation was repeated three times while decreasing the side of each interrogation square from 64 pixels to 32 pixels to 16 pixels, which allows to yield a higher vector resolution.

### Focused ion beam scanning electron microscopy (FIB/SEM) imaging of an evaporative deposit

An evaporative deposit for ${\varphi }_{{\rm{suc}}}=200$ mM was coated with osmium by osmium plasma coater (POC-3; Meiwa Shoji Co., Tokyo, Japan) and milled by focused Ga ion beam (30 kV, 0.75 A). The milled cross-section was imaged by SEM (Helios G4 UX, FEI, USA) at an acceleration voltage of 1.0 kV.

### Height measurements of evaporative deposits

Height profiles of evaporative deposits were measured using optical profilometer (OPTELICS HYBRID, Lasertec), equipped with ×50, 0.95 NA and ×100, 0.95 NA dry objective lens (Nikon, Japan). In the measurements, white light from a Hamamatsu xenon lamp (L8253) was used. The image processing and visualization after the measurements were done with the softwares (LMeye7 and SPIP). The inclination was corrected with three points on the glass substrate. Then, the noise was removed using the 2D median filter with the window size 3 × 3.

## Electronic supplementary material

Supplementary infomation

Supplementary movie 1

Supplementary movie 2

Supplementary movie 3

Supplementary movie 4

Supplementary movie 5

Supplementary movie 6

Supplementary movie 7
