# Supplementary material for: Suppression of the coffee-ring effect by sugar-assisted depinning of contact line
Source: Sci Rep. 2018 Dec 11;8:17769. doi: 10.1038/s41598-018-35998-w (PMC6289994; doi:10.1038/s41598-018-35998-w)
Supplement: Supplementary file 1 — Supplementary infomation [file 41598_2018_35998_MOESM1_ESM.pdf]

Supporting Information for Suppression of the coffee-ring effect by sugar-assisted depinning of contact line

Shunsuke F. Shimobayashi, Mikiko Tsudome and Tomo Kurimura

November 7, 2018

1 Supplementary Figures

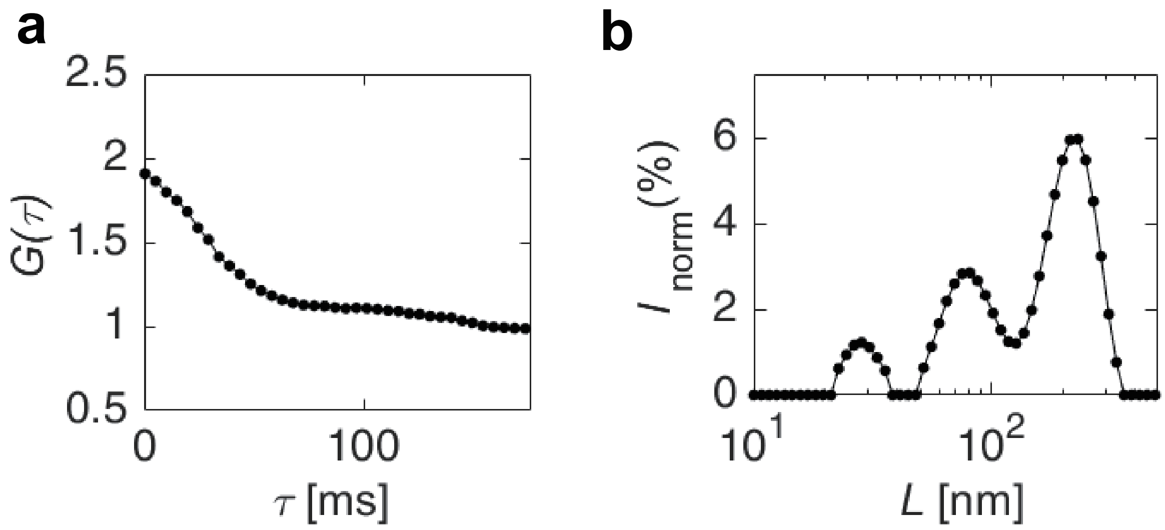

Figure 1: Particle size in coffee drops measured by dynamic light scattering. (a) Second-order time correlation function, G(τ), as a function of time delay τ, which was obtained from the light scattered by particles. The measurement was performed for 120 seconds using Otsuka FPAR-1000 system (Japan). The scattering angle was 160°. (b) Normalized scatted intensity, I_{norm}, as a function of the hydrodynamic radius L, which was obtained by CONTIN analysis of G(τ), in which three different peaks are confirmed, namely 29.0 ± 4.5 nm, 83.7 ± 15.4 nm, and 206.7 ± 43.2 nm, respectively.

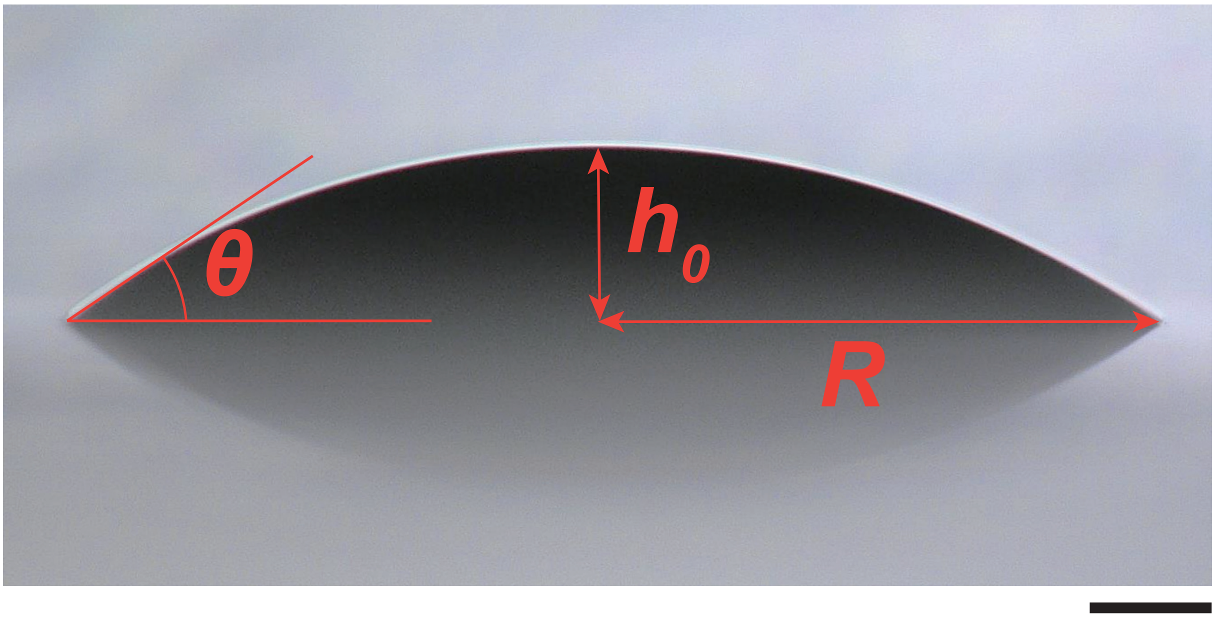

Figure 2: Initial shape of a drop for $\phi_{\mathrm{suc}} = 200$ mM on a glass substrate, which was observed from the side using VHX6000 (KEYENCE, Japan) equipped with objective lens (VH-ZST, KEYENCE, Japan). The parameters h_0, R, and θ were estimated by the image analysis. The scale bar is 200 μm.

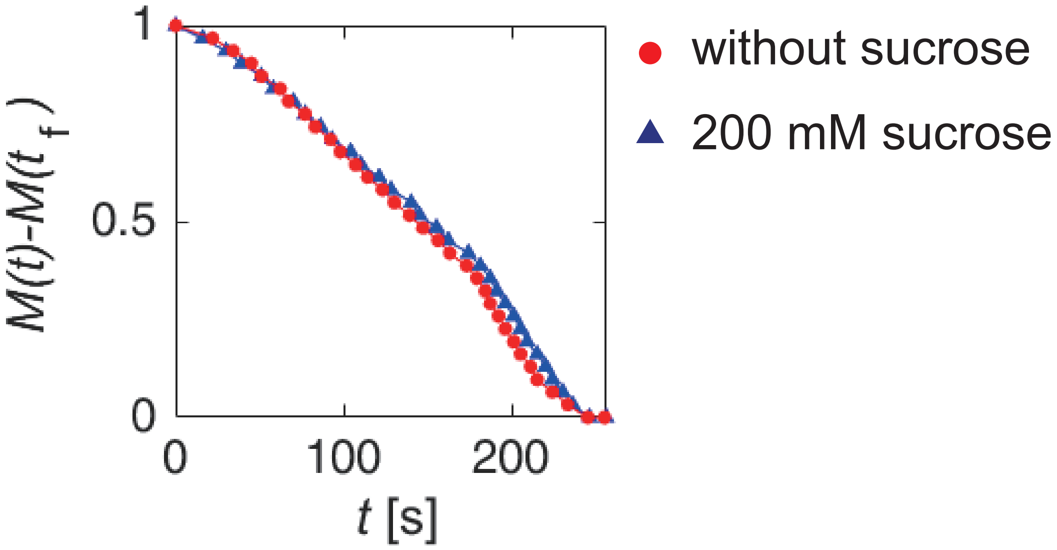

Figure 3: Normalized mass loss by evaporation, $M(t) - M(t_{\mathrm{f}})$, versus time t, where $M(t)$ and $M(t_{\mathrm{f}})$ are the mass measured at time t and final mass after evaporation. For the measurement, the electric balance (AG135 DualRange, METTLER TOLEDO, USA) was used.

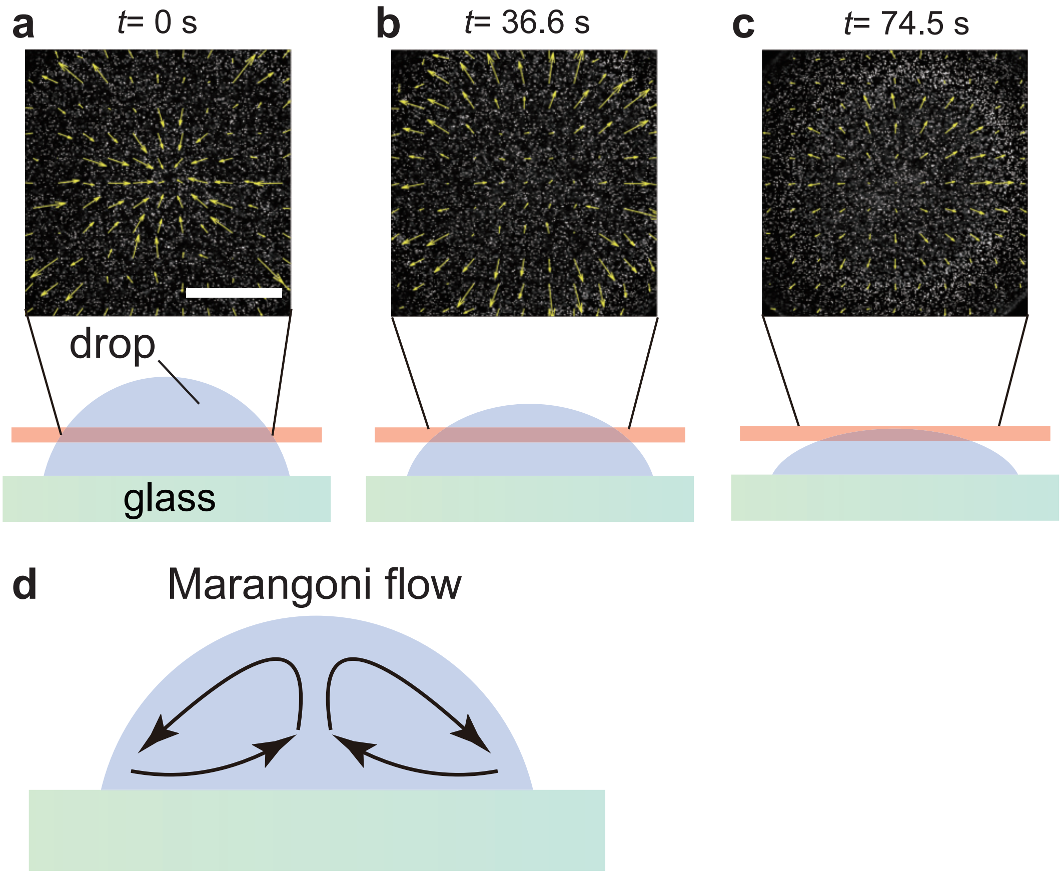

Figure 4: Flow field of an evaporating drop with $\phi_{\rm suc} = 200$ mM, which was estimated by PIV analysis at the height of 70 ± 1 μm from the bottom. (a) Flow filed at $t = 0$ s. The flow direction is inward and outward around the centre and edge, respectively. (b) Flow field at $t = 36.6$ s. The flow is nearly zero around the centre, and the flow direction is outward around the edge. (c) Flow field at $t = 74.5$ s. The flow direction at the top of the drop is outward. The focal plane is represented as the red-shaded region. The scale bar is 500 μm. See also the supplementary Movie 6. (d) Expected Marangoni eddies in the evaporating drop. The flow field is indicated by the arrows.

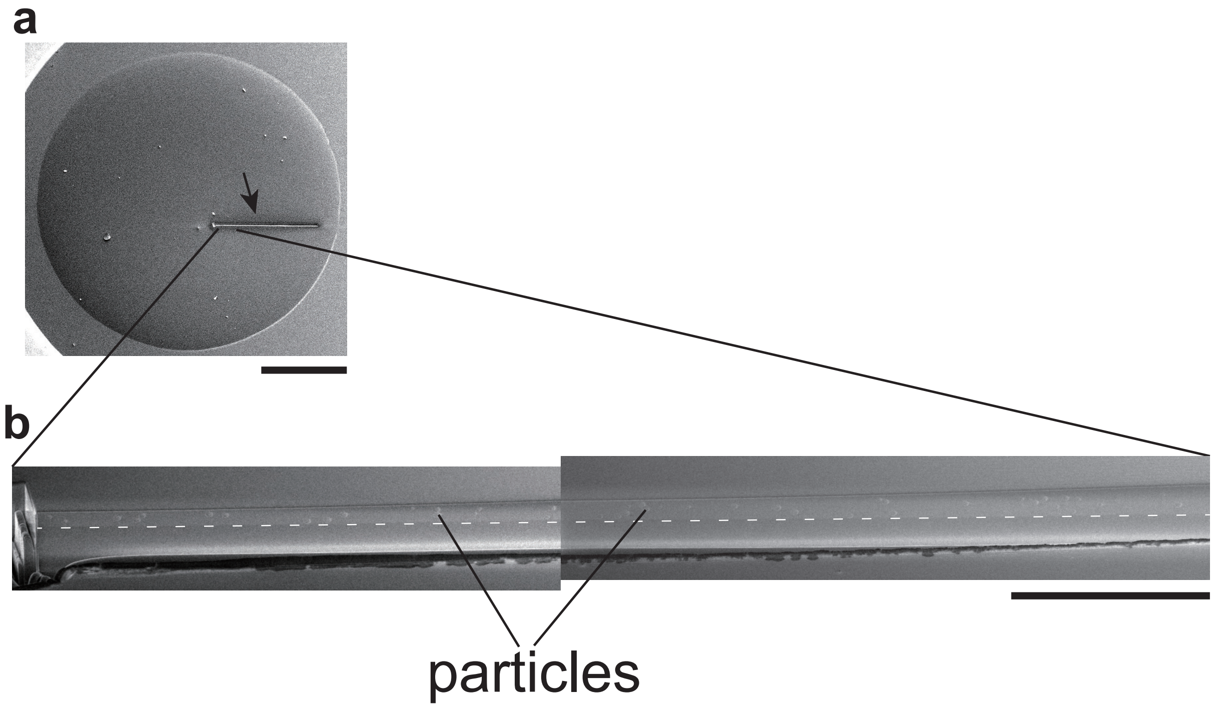

Figure 5: Cross sectional SEM images of the evaporative deposit of a drop with $\phi_{\text{suc}} = 200$ mM. (a) SEM image from the top. The arrow is a region milled with focused galium ion beam. The scale bar is 500 μm. (b) Cross sectional SEM images of the deposit. Particles are confined only above the white-dashed line. The scale bar is 30 μm.

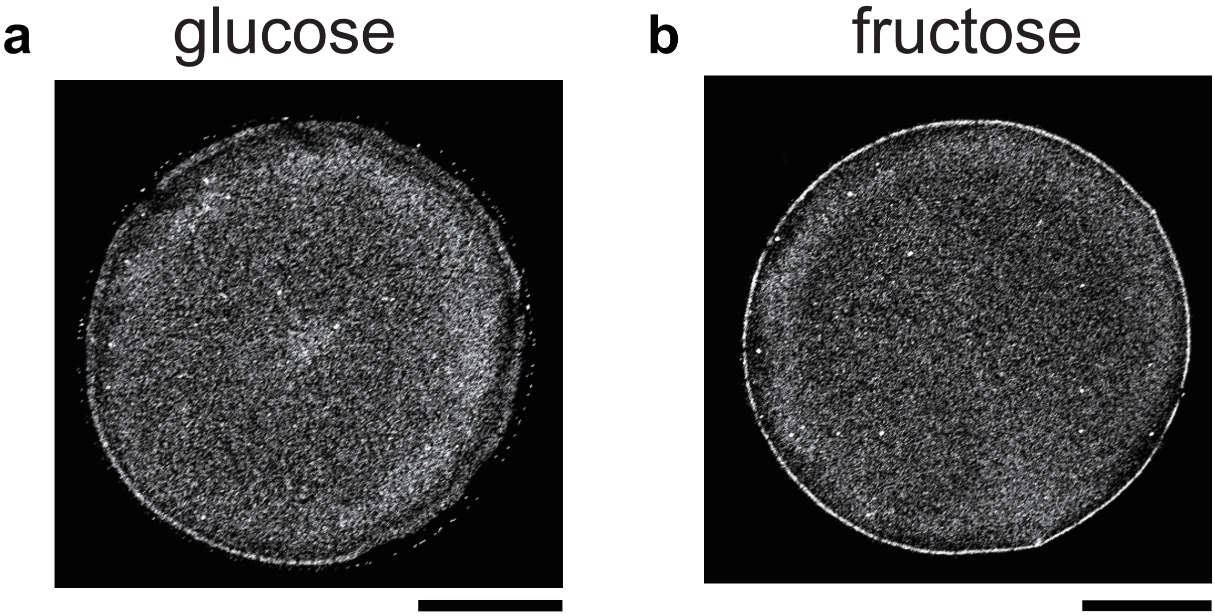

Figure 6: Evaporative deposits of a drop (0.5 μL, 0.01 vol%) of 1 M glucose (Wako, Japan) and 0.5 M fructose (Wako, Japan). The scale bars are 500 μm.

2 Captions of Supplementary Movies

Movie S1.

The movie shows an evaporating suspension of fluorescence particles with a sucrose concentration of 0.1 mM. Particles are transported to the triple line, leading to a ring-like deposit.

Movie S2.

The movie shows an evaporating suspension of fluorescence particles with a sucrose concentration of 100 mM. Particles are captured by the substrate due to the depinning of the triple line, leading to an uniform deposit.

Movie S3.

The movie shows an evaporating suspension of fluorescence particles with a sucrose concentration of 100 mM at a higher magnification. Particles at the edge of the droplet move towards the centre and are finally fixed.

Movie S4.

The movie shows the PIV results of an evaporating suspension of fluorescence particles with a sucrose concentration of 1.0 mM. The analysis was done near the glass substrate. The outward capillary flow rapidly grows at $t/t_\mathrm{f} \sim 0.9$, leading to a ring-like deposit.

Movie S5.

The movie shows the PIV results of an evaporating suspension of fluorescence particles with a sucrose concentration of 200 mM. The analysis was done near the glass substrate. The inward flow due to the solutal Marangoni flow is observed at the initial stage of evaporation. The final deposit is uniform.

Movie S6.

The movie shows the PIV results of an evaporating suspension of fluorescence particles with a sucrose concentration of 200 mM. The analysis was done at the height of 70 ± 1 μm. See supplementary Figure 4 for the details of flows. The final deposit is uniform.

Movie S7.

The movie shows an evaporating suspension of fluorescence particles with a sucrose concentration of 10 mM. The deposit shows an intermediate pattern because the recession of the triple line stops due to the absence of the solid-like sucrose.
